# Progressive chronic tissue loss disease in *Siderastrea siderea* on Florida’s coral reef

**DOI:** 10.1371/journal.pone.0329911

**Published:** 2025-08-06

**Authors:** Greta Smith Aeby, Yasunari Kiryu, Jan Landsberg, Blake Ushijima, Scott Jones, L. Jay Houk, Joseph Kuehl, Valerie J. Paul

**Affiliations:** 1 Smithsonian Marine Station, Fort Pierce, Florida, United States of America; 2 Fish and Wildlife Research Institute, Florida Fish and Wildlife Conservation Commission, St. Petersburg, Florida, United States of America; 3 Department of Biology and Marine Biology, University of North Carolina Wilmington, Wilmington, North Carolina, United States of America; 4 Mote Marine Laboratory, Elizabeth Moore International Center for Coral Reef Research and Restoration, Summerland Key, Florida, United States of America; 5 Reef Renewal USA, Tampa, Florida, United States of America; University of the Ryukyus, JAPAN

## Abstract

Stony coral tissue loss disease (SCTLD) has devastated numerous species of corals across the Western Atlantic but one reef coral, *Siderastrea siderea*, displays unusual tissue loss lesions. We examined the dynamics of lesions in *S. siderea* from the cellular to the ecological level and compared the disease with SCTLD in other coral species. We tagged and monitored six *S. siderea* colonies with bleached lesions in Fort Lauderdale and 17 *S. siderea* colonies with purple lesions in the Florida Keys for 18 months. Lesions on most colonies showed progressive tissue loss with an average change in healthy tissue of +5.5% in Fort Lauderdale (some bleached lesions resolved) and −51.1% in the Florida Keys. Case fatality rate was zero for colonies within Fort Lauderdale and 5.9% for colonies in the Florida Keys. The disease remained on *S. siderea* throughout the study in the Florida Keys but fluctuated through time in Fort Lauderdale. Lesion morphologies and disease pathogenesis differed between regions which could be due to different disease agents, environmental co-factors, intrinsic differences among colonies or different stages of the same disease. *S. siderea* is known to be a species complex which might also explain differences in lesion morphologies and disease pathogenesis. Aquaria studies found *S. siderea* with lesions transmitted disease to *S. siderea* and *Orbicella faveolata* and that *S. siderea* was also susceptible to SCTLD*.* Unlike SCTLD in other species, treatment with antibiotics did not stop lesion progression in *S. siderea*. Histology on lesions indicated a disease process regardless of lesion morphology and was consistent with SCTLD. We cannot completely rule out SCTLD but based on the other components of disease pathogenesis (rate of tissue loss, lesion morphology, colony mortality, response to antibiotics) we conclude this could be a different disease, which we term *Siderastrea sidera* chronic tissue loss disease, consistent with accepted disease nomenclature.

## Introduction

Coral reefs are declining at alarming rates, and two major contributing factors are increased frequency of coral bleaching events [1] and disease outbreaks [2,3]. Currently, in the Western Atlantic, a major threat to coral reefs is a long-term disease outbreak termed stony coral tissue loss disease (SCTLD). Stony coral tissue loss disease was first reported on reefs in the Miami-Dade region of Florida in 2014 [4], subsequently spread throughout all of Florida’s Coral Reef and then into the neighboring Caribbean islands [5]. SCTLD affects over 30 species of reef corals and has decimated numerous affected coral reefs [6–8]. Disease signs vary widely among coral species and tissue loss lesions on colonies can appear as focal or multi-focal spots, with or without adjacent bleached margins. Gross lesions on corals cannot be used to diagnose a specific disease unless the disease agent is clearly visible, e.g., black band disease or ciliate disease [3,9,10]. This is especially true for SCTLD, which has a diversity of lesion morphologies and the etiology of SCTLD is not well understood, although studies have demonstrated that bacterial pathogens are involved [11–13]. SCTLD is usually suspected if there are multiple species of corals on a reef with progressive tissue loss lesions and the coral species known to be most susceptible to SCTLD are affected. Most coral species exhibit an acute to subacute rate of tissue loss from SCLTD, which is based on the degree of denuded white coral skeleton visible on the colony, however, some species have a slower, chronic rate of tissue loss [14]. Regardless of the gross lesion morphology, most histopathology on suspected SCTLD cases within Florida, find a hallmark microscopic lesion with lytic necrosis originating in the gastrodermis of the basal body wall and degraded symbiotic zooxanthellae [14].

The reef coral, *Siderastrea siderea*, does display tissue loss lesions consistent with SCTLD [15–17] but also presents with unusual lesions with multi-focal bleached spots, spots of purple discoloration (pigmentation response) and presenting with or without associated tissue loss. Pigmentation in corals is a general stress response which can be due to pathogens, invasive organisms, or indicative of general environmental stress [18,19]. So, the question remains as to whether this species is affected by the same presumptive pathogen(s) as the other coral species affected by SCTLD, but disease signs vary or if the lesions with discolorations (bleaching or purple) are a different disease altogether. Complicating this question is the finding that *S. siderea* is likely a species complex, with different lineages being reported among samples collected in Florida [20] and Panama [16]. *Siderastrea siderea* is known to be comparatively resistant to environmental stressors and as such, *S. siderea* densities have been increasing through time as other historically dominant species decline [21–23]. For example, *S. siderea* is resistant to bleaching due to high irradiance or temperature stress (hot or cold) and readily recovers from temperature stress events [24–26]. Six diseases have been reported affecting *S. siderea* [27], but only dark spot disease (dark spot syndrome) has been well studied [18,28–31] making it difficult to interpret lesions in this species. To aid in understanding disease in *S. siderea*, we examined disease dynamics of lesions in *S. siderea* from the cellular to the ecological level and compared the disease ecology with other coral species diagnosed with SCTLD. Our objectives were to: 1) examine lesion morphologies and subsequent disease virulence (rates of tissue loss) by following tagged colonies with lesions through time, 2) examine the histopathology of lesions, 3) conduct aquaria studies to determine whether *S. siderea* lesions are transmissible and 4) whether *S. siderea* is susceptible to SCTLD, and 5) use antibiotics to determine whether bacteria might be involved in the disease process.

## Materials and methods

### Study sites

We tagged *S. siderea* colonies at two sites, Fort Lauderdale and the Florida Keys. The Fort Lauderdale site is located offshore of Broward County in southeast Florida. The reef system in this region is a northern continuation of the Florida reef tract. The site (26.14858ºN 80.09591ºW) is located approximated 0.5 km from shore at approximately 8 meters depth and was used for previous studies on tagged colonies with SCTLD [11,29]. The other site (Haslun’s Reef) was in the Florida Keys located within the Looe Key Existing Management Area (https://floridakeys.noaa.gov/zones/spas/looekey.html). Haslun’s Reef (24.55234ºN 81.43745ºW) is approximately 6.7 meters in depth. A nearby site within Looe Key (24.54599^◦^N, 81.40400^◦^W) was also used for previous tagging studies [32]. Looe Key is a popular dive site within the Florida Keys National Marine Sanctuary that is visited by hundreds of thousands of snorkelers and divers each year (https://floridakeys.noaa.gov/).

### Lesion morphologies and mortality through time among diseased *S. siderea* colonies in the Florida Keys and Fort Lauderdale regions

**Fig 1:**
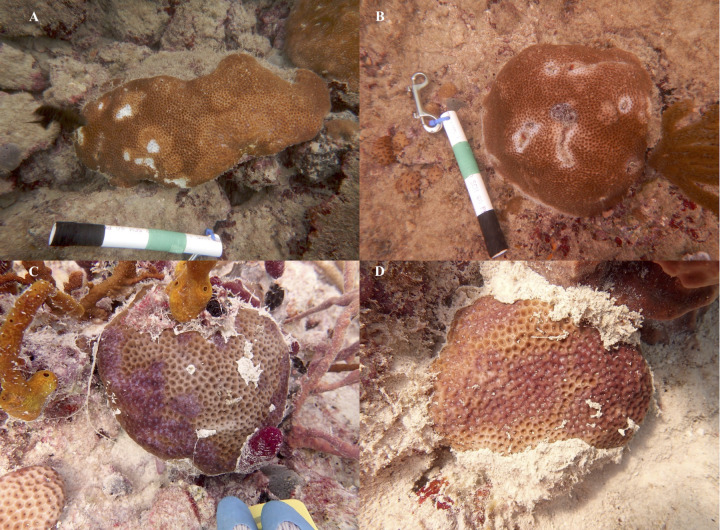
Gross lesions on *S. siderea* colonies Examples of *S. siderea* lesions on colonies tagged in Fort Lauderdale (A and B) and Florida Keys (C and D). White areas on colonies in Fort Lauderdale indicates bleaching not tissue loss.

Six *S. siderea* colonies with lesions (multi-focal bleached patches) were tagged in the Fort Lauderdale region and seventeen *S. siderea* colonies with lesions (bleached, purple or dark discoloration with or without associated tissue loss) were tagged in the Florida Keys region (Haslun’s reef). Sixteen of the seventeen tagged colonies had purple or dark discoloration with or without tissue loss and a single colony had multi-focal bleached lesions (Fig 1). All colonies were initially tagged, mapped out on the reef, and photographed in November 2019 and then were photographed approximately every other month thereafter, or as possible until May 2021 (18 months). Colonies in the Florida Keys were photographed by Mote Marine Laboratory staff on Nov. 18, 2019, Jan. 15, March 23, May 27, July 27, Sept. 28, Dec 5, 2020, and May 6, 2021. Colonies at Fort Lauderdale were photographed by Broward County Environmental Protection and Growth Management Department personnel on Nov. 22, 2019, April 16, July 2, Oct 12, 2020, and Jan 8, March 4, and May 27, 2021. Changes in lesion morphology and/or colony mortality were assessed via photograph review following [11,32]. The multidimensional structure of the colonies and the presence of multi-focal lesions complicated the use of digital image analysis for calculating the rates of lesion progression. Hence, a semi-quantitative estimate of lesion progression was used whereby visual estimates of colony health (proportion of colony healthy, old dead, or lesioned (pigmented or recent tissue loss) were assessed from photo review by G.A. Recent tissue loss was indicated by the presence of bare, white skeleton and “old dead” were tissue loss areas covered in sediment, algae or other organisms. Total tissue loss was determined by visually estimating the proportional loss of healthy tissue between the starting (Nov 2019) and ending (May 2021) study periods. Data did not have a normal distribution, so a non-parametric Wilcoxon two-sample test was used to examine differences in total tissue loss (%) among lesion types within the Florida Keys. All statistics were completed using JMP^®^ Pro 18.

### *Siderastrea siderea* histopathology

Samples from sixteen *S. siderea* colonies with lesions were collected and preserved in 1 part Z-Fix concentrate (Anatech Ltd. Battle Creek, MI) mixed with 4 parts natural seawater. These 16 samples comprised diseased specimens exhibiting diverse lesions from two regions along Florida’s coral reefs. Within the Florida Keys, eight samples were collected from Looe Key (24.54599°N 81.404°W) on November 18, 2019, and five from Haslun’s Reef (24.55234°N 81.43745°W) on November 19, 2019. Three samples were collected from Fort Lauderdale (26.148585°N 80.095915°W) on November 22, 2019. Additionally, six apparently healthy reference coral samples were collected from three sites within the Dry Tortugas (site 1: 24.59346°N 82.98103°W; site 2: 24.64273°N 82.96935°W and site 3:24.60526°N 82.96913°W) between January 28–29, 2020 before SCTLD was recorded in the region.

Post-fixed samples were transported to FWC/FWRI at St. Petersburg, FL for processing with routine paraffin embedded histological specimens. For ease of orientation and maintaining coral tissue integrity, samples were first enrobed with 1.5% agarose to hold tissues and associated surface biota in place after the skeleton was removed following decalcification processing with 10% ethylenediaminetetraacetic acid solution (Fisher Scientific). Decalcification required an average of 49.7 days (SD ± 19.7 d) (n = 22).

Decalcified tissues were organized for sectioning orientation at both radial (cross, parallel to the polyp mouth) and sagittal (longitudinal, perpendicular to the polyp mouth) angles. Routine paraffin embedded histologic sections were cut at four µm, stained with Mayer’s hematoxylin and eosin (H&E) and thionin stains [33]. Tissues were also embedded with glycol methacrylate plastic resin (JB-4; Electron Microscopy Sciences, Hatfield, PA) with arbitrary angle, sectioned at 4.0 µm, and stained with Weigert’s H&E, thionin, and periodic acid–Schiff–metanil yellow (PAS-MY) [34]. Histological parameters recorded were as follows: lytic necrosis occurring at the gastrodermis, coagulative necrosis in the general tissues, changes in the symbiotic microalgae Symbiodiniaceae (such as PAS reaction, hypertrophy, atrophy, necrosis, symbiosome condition), abundance of coral-acid rich protein (CARP) granules at the surface area, and mucus abundance. The presence of other associated organisms, such as endolithic sponges or algae, were noted.

### Transmission from diseased *Siderastrea siderea* to healthy *S. siderea* and *Orbicella faveolata*

**Fig 2:**
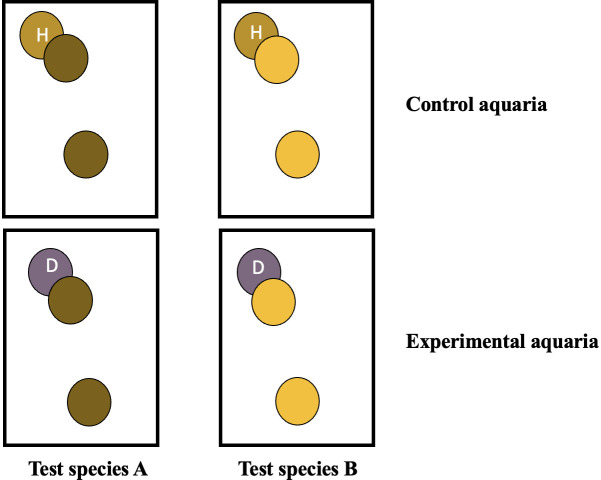
Schematic of the experimental design for transmission studies in aquaria. Symbols with the same color indicate coral fragments were collected from the same colony. ‘H’ refers to an apparent healthy fragment and ‘D’ refers to a disease fragment displaying lesions.

Aquaria trials were used to determine whether lesions in *S. siderea* were transmissible and whether there were differences in susceptibility between *S. siderea* and *O. faveolata*. For each experimental block, there were two aquaria (experimental and control) used for each test species and two test species (*S. siderea* and *O. faveolata*) used in each trial for a total of four aquaria per block (Fig 2). To test for direct transmission, a fragment of *S. siderea* with a distinct lesion was placed in direct contact with a healthy fragment and to test for waterborne transmission, the other healthy fragment was placed ~10 cm away. To test for lesions created by coral-to-coral aggressive interactions, control aquaria were set up in the same manner except the diseased *S. siderea* fragment was replaced with a healthy fragment of *S. siderea*. Fragments with lesions were cut in half with a rock saw (decontaminated with 80% ethanol between use on healthy and diseased corals) and used for the comparative study between intra- and inter-specific rates of transmission, ensuring that each test species (*S. siderea* or *O.* f*aveolata*) was exposed to a similar level of potential infectiousness from the lesions on *S. siderea* fragments. Any corals that developed lesions in the experimental or control tanks were removed from contact and observed until the end of the experiment. Lesions that progressed after contact was removed were considered probable disease transmission but lesions that did not progress or started to heal suggested that lesion formation was due to coral-to-coral aggression. All experiments were conducted in outdoor tanks at the Smithsonian Marine Station under natural light with shade cloth used to reduce ambient light by ~50%. Aquaria were maintained in larger water tables with circulating freshwater adjusted with a cooling and heating system to maintain aquaria water temperatures between 28–29°C. Aquaria were filled with seawater filtered through a 0.22 μm filter (FSW) and a bubbler placed in each aquarium to create water motion. Fragments were photographed each day and partial water changes done daily to maintain water quality. The source of seawater used in all experiments was filtered, offshore ocean water described in [35]. Fragments used in all experiments were collected from Florida’s reefs but as coral is currently a limited resource on these reefs, priority was given to using nursery corals or corals already growing in water tables at the Smithsonian Marine Station. This minimized coral collected directly from the coral reefs. Hence, experimental coral fragments were in captivity anywhere from days to months before the experiments. All fragments with disease lesions, used in the experiments, were collected from the field and used within a day or two after collection to avoid tissue loss from potentially rapid disease progression. To meet assumptions of independence among aquaria sets (set = experimental and control) care was taken to ensure that all apparently healthy fragments and fragments with disease lesions were from different colonies. The experiment was conducted for a maximum of 21 days. Eleven sets of aquaria (blocks) were tested in October 2018 and an additional 7 sets tested in May 2021 (total = 18). A Mantel–Cox log rank test was used to examine species differences in rate of infection in the transmission trials. Touching and non-touching fragments were analyzed separately. A Mantel-Cox log rank test was also used to examine differences in rate of transmission among lesion types (discoloration versus discoloration with associated tissue loss). For this comparison, *S. siderea* and *O. faveolata* were analyzed separately and only the fragments tested with direct transmission were considered. A Mantel– Cox log rank test is a non-parametric test that compares the survival distributions of two groups and examines the outcome of the trial (transmission or no transmission) and the time to the outcome. All statistics were completed using JMP^®^ Pro 18.

### Transmission from diseased (apparent SCTLD) *Colpophyllia natans* to healthy *S. siderea*

We conducted aquaria studies to directly examine the susceptibility of *S. siderea* to acute tissue loss lesions on *C. natans* indicative of SCTLD. Experiments were conducted using two aquaria per set (experimental and control). In the experimental tanks, an infected fragment of *C. natans* with an acute tissue loss lesion was placed in direct contact with a healthy fragment of *S. siderea* (direct transmission), and another healthy fragment of *S. siderea* was placed ~10 cm away (waterborne transmission). In the control aquaria, the fragment with tissue loss lesions was replaced with a healthy fragment of *C. natans* to control for lesions created by coral-to-coral aggressive interactions. Healthy test corals were cut into four pieces ensuring the same test genotype in all aquaria sets. To discriminate between lesions caused by aggression versus a transmissible disease, any corals that developed lesions in the experimental or control tanks were removed from contact and observed for signs of lesion progression or recovery until the end of the study. Lesions that progressed following removal from contact were considered indicative of disease transmission. Lesions that failed to progress or healed were considered indicative of coral-to-coral aggression. Trials were held at the Smithsonian Marine Station under the same temperature-controlled conditions using filtered seawater as described above. Fragments were photographed daily, and partial water changes conducted to maintain water quality. The experiment was conducted for a maximum of 10 days. One set of aquaria were run in November 2018 (n = 3) and a second set in June 2019 (n = 2) for a total of five experimental sets.

### Therapeutic diagnosis with antibiotic treatment

A therapeutic diagnostic approach using antibiotics was taken to test for the possible role of bacteria in lesion progression with 18 pairs of diseased *S. siderea.* A *S. siderea* fragment with a lesion was cut in half using a rock saw so that each coral fragment had a relatively equal area of the lesion (experimental and control). The experimental fragments were treated with antibiotics resuspended in filtered aquarium water and the other fragment from each pair was left untreated as a control. Every fragment was individually housed in an aquarium with 0.22 μm filtered seawater (FSW) and a bubbler for water motion. Both experimental and control aquaria were placed in larger water tables with circulating freshwater adjusted with a cooling and heating system, to maintain water temperatures within aquaria between 28–29 °C. Coral fragments were photographed daily, and partial water changes were conducted every other day to maintain water quality. A complete water change was conducted weekly. For the experimental fragments, FSW was replaced with FSW pre-mixed with a combined treatment of amoxicillin (final concentration 50 μg/ml of tank water) and kanamycin (final concentration 50 μg/ml of tank water) consistent with prior studies on SCTLD [11]. Experiments with 11 pairs of fragments were conducted in October 2018 and maintained for a maximum of 4 weeks. The experiment was repeated in June 2019 on 7 pairs of fragments using the same methods with the exception that corals were dosed daily with antibiotics and the trial was run for 2 weeks. The outcome of the experimental exposure to antibiotics was the same in 2018 and 2019 so the data were combined for analysis. To examine the effect of antibiotic treatment on lesion progression, a McNemar’s test for paired nominal data was used. McNemar’s test compared the outcome (lesion progression or not), after treatment for the two groups (antibiotic and control). Test fragments were scored as positive or negative for lesion progression.

## Results

### Lesion morphologies and mortality through time among diseased *S. siderea* colonies in the Florida Keys and Fort Lauderdale regions

**Fig 3:**
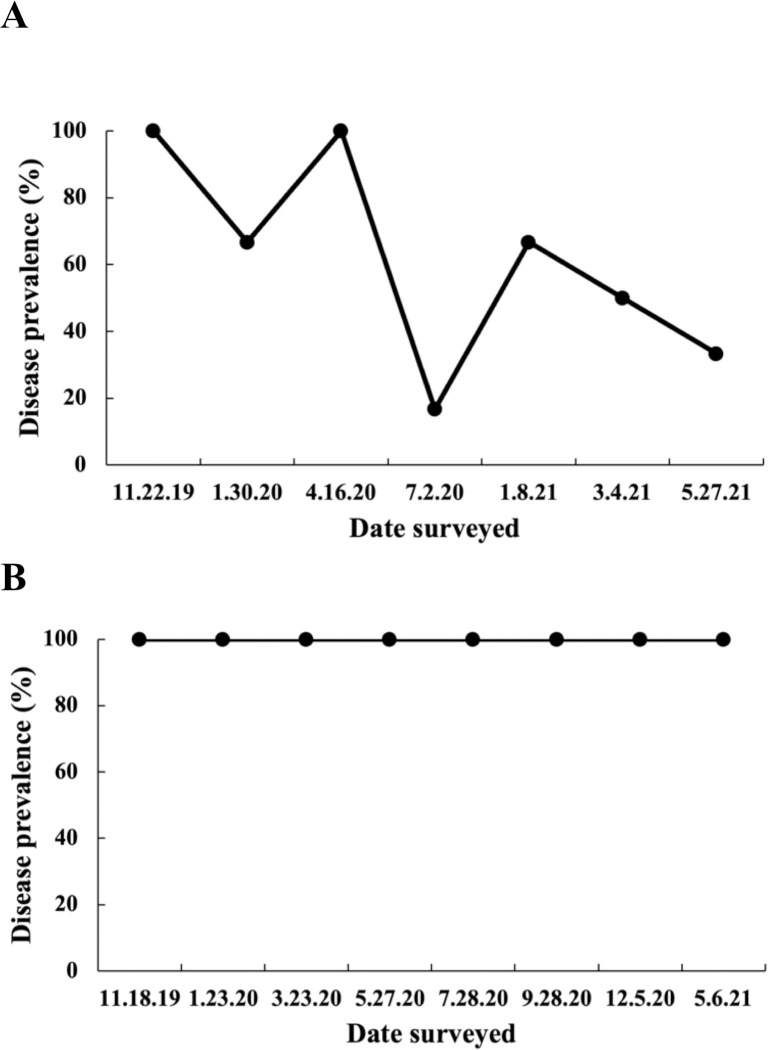
Regional differences in disease prevalence through time for tagged colonies of diseased *S. siderea.* (A) Prevalence of six colonies in Fort Lauderdale. (B) Prevalence of 17 colonies in the Florida Keys.

At the Fort Lauderdale site, all six *S. siderea* colonies initially had lesions (prevalence = 100%) but through time disease incidence fluctuated between 16.7% and 100%, whereas at the Florida Keys site, all 17 *S. siderea* colonies that initially had lesions (prevalence = 100%) remained diseased throughout the 18-month study period (Fig 3). Case fatality rate (total colony mortality) was low for both regions. There were no fatalities in Fort Lauderdale (0%) and only one out of the 17 colonies died in the Florida Keys (5.9%).

**Fig 4:**
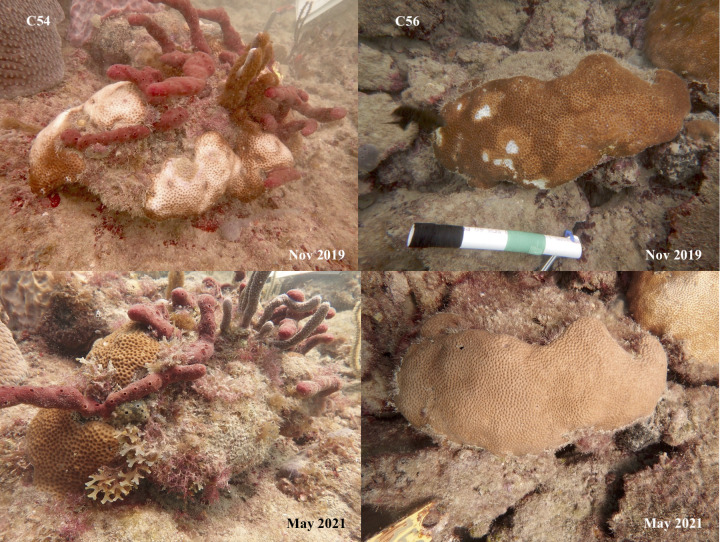
Representative photos of tagged *S. siderea* with lesions at Fort Lauderdale. C54 (left) shows a bleached lesion that progressed to partial tissue loss. C56 (right) shows a bleached lesion that resolved through time.

**Fig 5:**
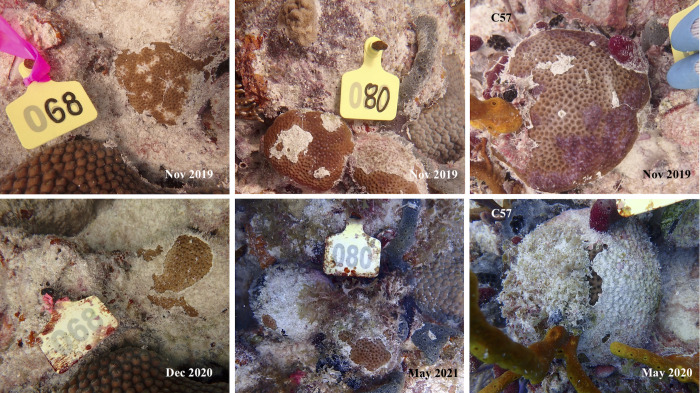
Representative photos of tagged *S. siderea* with lesions in the Florida Keys. C68 (far left panel) shows a colony with initial multi-focal bleached patches with some tissue loss (Nov 2019) that shows lesion resolution (no bleached spots) but with partial colony mortality (Dec 2020). C80 (middle panel) shows two colonies with initial tissue loss lesions and purple discoloration (Nov 2019) with both colonies progressing to near complete colony mortality (May 2021). C57 (far right panel) shows colony with initial multi-focal purple discoloration with some tissue loss (Nov 2019) that progresses to near complete mortality (May 2020).

At Fort Lauderdale, initially all colonies had bleached lesions with five of six colonies having no initial signs of tissue loss. The average change in healthy tissue was + 5.5% (range = −50% to +100%) with two colonies showing progressive tissue loss, one colony remaining stable (no change) and three colonies showing signs of lesion regression (re-pigmentation) (Fig 4). In the Florida Keys, initially lesions were either discolorations (purple or dark brown) without tissue loss (35.3% of the tagged colonies) or discoloration (purple, dark brown or bleaching) with some tissue loss (64.7%). Overall, the average amount of healthy tissue declined by 51.1% (range = +100% to −100) which is approximately 2.8% tissue loss/month (Fig 5). There were significant differences in total amount of tissue loss depending on initial lesion type (Wilcoxon 2-group test, Z = 1.97, P = 0.048). Colonies with discolored lesions initially lacking associated tissue loss (n = 6) had an average loss in healthy tissue of 9.9% (range = −100% to +100%), whereas colonies with discolorations with any initial signs of tissue loss (n = 11) lost an average of 69.2% (range = −12.7% to −100%). Lesion morphologies changed through time and by the end of the study all colonies had signs of chronic tissue loss with 10 out of the 17 colonies (58.8%) having mild bleaching adjacent to the lesions, five colonies (29.4%) had mild purple or brown discoloration adjacent to lesions and two colonies had no adjacent tissue pigmentation response (11.8%).

### *S. siderea* gross observations and histopathology

**Fig 6:**
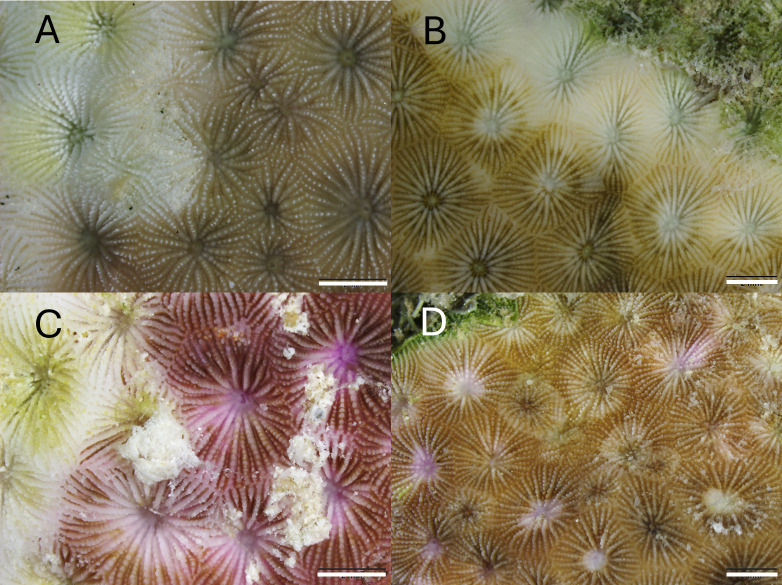
Gross pathological changes of post-fixed *S. siderea* collected from Florida’s Coral Reef. (A) tissue loss lesions with no pigmentation. (B) tissue loss with bleached lesions. (C) tissue loss with purple discoloration. (D) purple discoloration. All scale bars = 2 mm.

**Fig 7:**
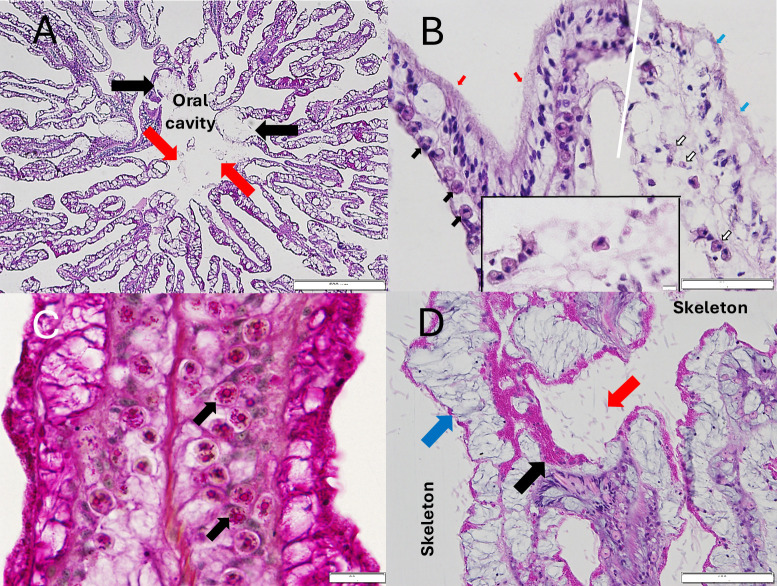
Histopathology of *S. siderea* collected from Florida’s Coral Reef. (A) Radial section across the oral cavity showing lytic necrosis lesions (black arrows) and sloughing of necrotic tissue into the oral cavity (red arrows) (H&E; scale bar = 500 µm). (B) Representative sagittal section along the septa showing border (white line) between surface bleaching (blue arrows) with reduced density of, loss of, or *in situ* necrosis of endosymbiotic Symbiodiniaceae with abnormal appearance (white arrows), and apparently healthy tissue (red arrows) with endosymbionts (black arrows). Note abnormal appearance of remnant endosymbionts (white hollow arrows) and general loss of cytoplasm or increased mucus (white space) in lesioned area (H&E; scale bar = 50 µm). Inset shown with enlarged abnormal endosymbiont (scale bar = 20 µm). (C) Periodic acid-Schiff stain (PAS) Sagittal section at the gastrodermal layer close to the surface area showing endosymbionts filled with PAS-positive (pink, red) starch granules (black arrows) (PAS-MY: scale bar = 5 µm). (D) Sagittal section of the mesenterial filament close to the surface area showing prominent coral-acid rich protein (CARP) granules (black arrow) in the calicodermal layer, possibly reacting to adjacent endoliths (red arrow) in the skeleton. Compare with apparent (subjective) thinner layer or presumptive lower density of CARPs (blue arrow) on the other side of the mesentery with less endoliths (H&E; scale bar = 500 µm).

Sixteen colonies with lesions were sampled for histology of which 31.3% had tissue loss lesions with no adjacent pigmentation (Fig 6A), 12.5% had bleached lesions with tissue loss (Fig. 6B), 37.5% had purple/brown discoloration with tissue loss (Fig 6C), and 18.7% had purples discoloration with no tissue loss (Fig 6D) (S1 Fig). Of 16 histology sections of lesions from these colonies, 13 had lytic necrosis (Fig 7A) (81.3%), 10 also had coagulative necrosis (62.5%) and 13 had intracellular, symbiotic Symbiodiniaceae with signs of degradation (81.3%) (Fig 7B; Table 1;S1 Table). Symbiodiniaceae with intracytoplasmic periodic acid-Schiff (PAS)-positive materials (starch) were seen only at the Looe Key site (Fig 7C; 62.5%) but were not found at Haslun’s Reef or Fort Lauderdale (0%) (Table 1). Coral-acid rich protein (CARP) granules in the calicodermis were generally present in all specimens examined (characteristically present in *S. siderea*, and notably eosinophilic in H&E), but multifocally, prominently aggregated CARPs (Fig 7D) were found in the diseased specimens (87.5%) (Table 1). CARPs potentially indicate a host response to endolithic invasion. Endolithic algae and fungal hyphae were found in all affected *S. siderastrea* from all sites (100%) (Table 1). However, the fungal hyphae never invaded coral tissues, that is the hyphae did not penetrate the calicodermis, mesoglea, and basal gastrodermis.

**Table 1:** Percent of microscopic lesions observed in histology sections of *S. siderea* collected in different regions along Florida’s Coral Reef.

Collection site (sample #)	LN^a^	CN^b^	Symbionts^c^ degradation	Symbionts^d^ PAS + Rx	CARPs^e^	Endoliths^f^
Looe Key (n = 8)	62.5	87.5	62.5	62.5	100	100
Haslun’s reef (n = 5)	100	40	100	0	80	100
Fort Lauderdale (n = 3)	100	33.3	100	0	66.6	100
**Total diseased (n = 16)**	**81.3**	**62.5**	**81.3**	**0**	**87.5**	**100**
Control (Dry Tortugas) (n = 6)	0	0	0	0	0	0

^a^Lytic necrosis

^b^Coagulative necrosis

^c^Symbiodiniaceae with signs of degradation

^d^Symbiodiniaceae with Periodic acid-Schiff positive reaction

^e^Coral acid rich proteins aggregates

^f^Algae hyphae

Healthy reference samples obtained from the Dry Tortugas exhibited no tissue necrosis nor observable degradation of the Symbiodiniaceae. Symbiodiniaceae at the surface gastrodermis had a brownish to reddish cytoplasm (under H&E), with a solid homogeneous appearance, and exhibited a PAS-negative reaction. Prominent endolithic algae and fungal hyphae were not found in the samples from Dry Tortugas (Table 1). Furthermore, CARPs were barely present or were less dense in the calicodermal tissue, especially those located just below the surface.

Additional observations were noted. Sponges, some of which were degraded (along with spicules and sometimes associated with eosinophilic granulocytes) were detected in the skeletal tissue, especially at the aboral region and were found at Looe Key (37.5%; n = 3) and Haslun’s Reef (80%; n = 4). Only one sample from Haslun’s Reef had a clionid (a boring sponge with Symbiodiniaceae) near the septal surface and in the basal area of the skeletal tissue.

### Transmission from diseased *S. siderea* to healthy *S. siderea* and *Orbicella faveolata*

**Fig 8:**
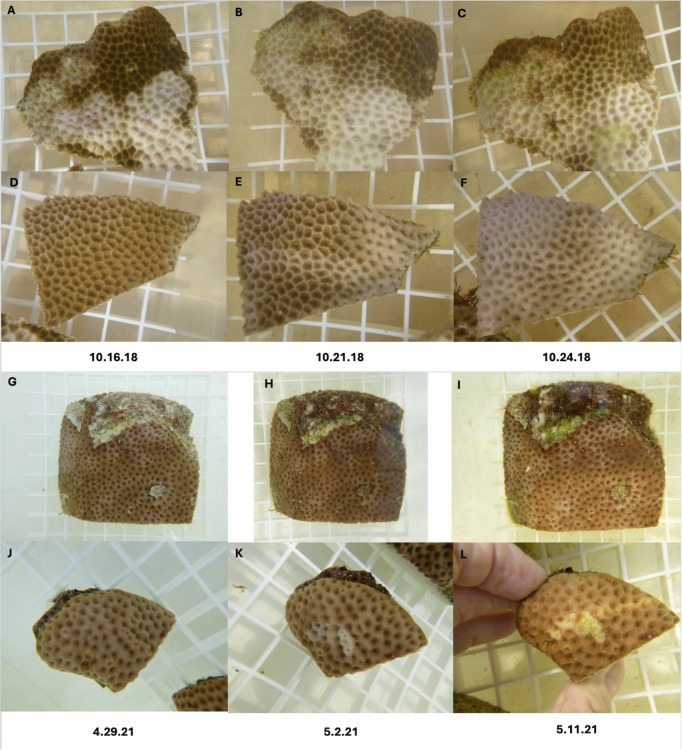
Representative photos of disease progression in coral fragments used in transmission experiments using *S. siderea.* A-C and G-I are diseased *S. siderea* and D-F and J-L are test fragments that developed lesions. Left panels shows the fragments at the start of the experiment. Middle panels show fragments on the date that contact was removed between healthy test fragments and the diseased *S. siderea*. Right panels show fragments on termination of the experiment. A-F shows transmission that resulted in bleaching. G-L shows transmission that resulted in progressive tissue loss.

**Fig 9:**
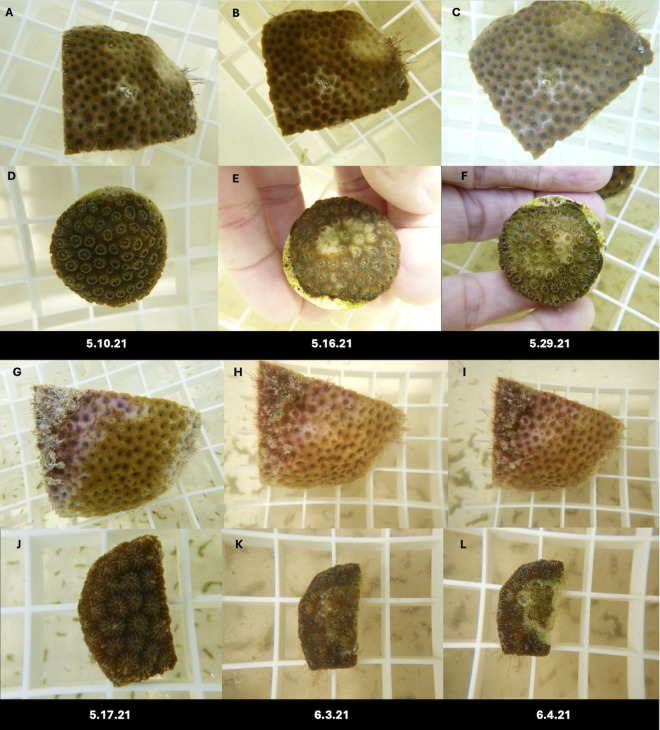
Representative photos of disease progression in coral fragments used in transmission experiments using *S. siderea* and *O. faveolata.* A-C and G-I are diseased *S. siderea* and D-F and J-L are test *O. faveolata* fragments that developed lesions. Left panels shows the fragments at the start of the experiment. Middle panels show fragments on the date that contact was removed between healthy test fragments and the diseased *S. siderea*. Right panels show fragments on termination of the experiment. A-F shows transmission from diseased *S. siderea* with a bleached lesion. G-L shows transmission from *S. siderea* with a lesion with purple pigmentation.

When visually healthy *S. siderea* and *O. faveolata* were exposed to *S. siderea* with lesions there was a similar rate of disease transmission (development of a lesion) for both species upon direct contact (Mantel-Cox log-rank test, X^2^ = 2.57, df = 1, p = 0.11) and through the water column (Mantel-Cox log-rank test, X^2^ = 1.97, df = 1, p = 0.16). Four of the 18 healthy *S. siderea* fragments (22.2%) touching a diseased *S. siderea* developed lesions after an average of 13.5 days (range = 8–12 days) and one of 18 non-touching *S. siderea* developed a lesion (5.6%) after 8 days. Transmission to *O. faveolata* was more successful with 9 out of 18 (50%) of the touching fragments developing a lesion after an average of 7.5 days (range = 2–19 days) and 3 of 18 (16.7%) of the non-touching fragments developing disease signs after an average of 7.5 days (range = 4–19 days). All new lesions, on touching fragments, originated from the point of contact between the experimental and test fragments, and lesions on all test fragments continued to progress after contact with the disease was discontinued (Figs 8 and 9). The control fragments also developed lesions from coral-coral aggression in five out of 18 (27.8%) *S. siderea* and 9 out of 18 (50%) of the *O. faveolata.* However, once contact between fragments was stopped then lesion progression stopped on all control fragments.

### Differences in direct transmission among lesion types

Based on our findings in the field on the tagged colonies, we categorized the initial lesions on the diseased *S. siderea* as purple or bleaching discoloration without initial tissue loss and purple, bleaching or no discoloration with tissue loss. We then calculated the overall transmission success with direct contact (2018 and 2021 experiments combined) among the two lesion types for each test species (*S. siderea* and *O. faveolata*). For *S. siderea,* 4 out of 11 fragments touching lesions with initial discoloration plus tissue loss developed lesions (36.4%) whereas none of the 7 fragments touching discolored lesions without initial tissue loss developed lesions (0%) (Mantel-Cox log-rank test, X^2^ = 0.00, df = 1, p = 0.99). For *O. faveolata*, seven out of 11 fragments touching discolored lesions with initial tissue loss developed lesions (63.6%) and 2 out of 7 fragments touching discolored lesions lacking tissue loss developed lesions (28.6%)(Mantel-Cox log-rank test, X^2^ = 0.02, df = 1, p = 0.88).

### Transmission from diseased (apparent SCTLD) *C. natans* to healthy *S. siderea*

When healthy *S. siderea* were exposed to *C. natans* with tissue loss disease, all 5 touching *S. siderea* (100%) developed tissue loss lesions after an average of 8.4 days (range 6–10 days) and 4 of the 5 (80%) non-touching *S. siderea* developed tissue loss lesions after an average of 7 days (range 6–8 days). Lesions in *S. siderea* fragments touching *C. natans* progressed after contact was discontinued. In control aquaria, all healthy *S. siderea* touching healthy *C. natans* also developed tissue loss lesions but all lesions began to heal once contact was terminated.

### Therapeutic diagnosis with antibiotic treatment

**Fig 10:**
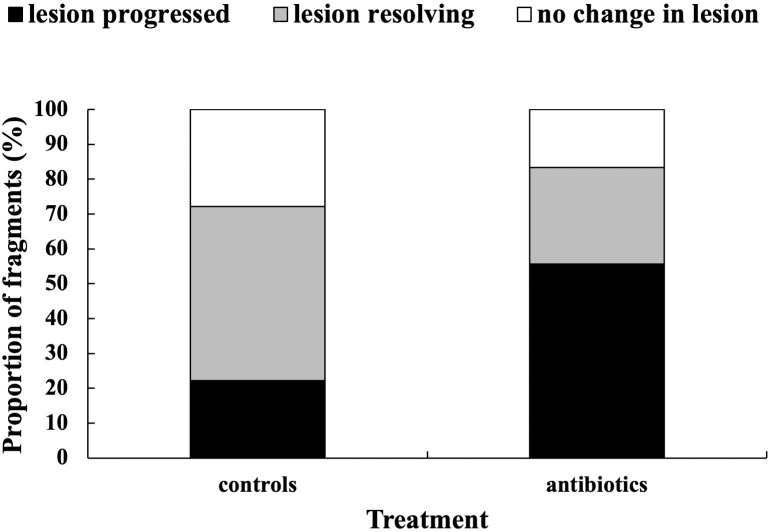
Response of *S. siderea* with lesions to treatment with antibiotics. (n = 18 paired fragments).

**Fig 11:**
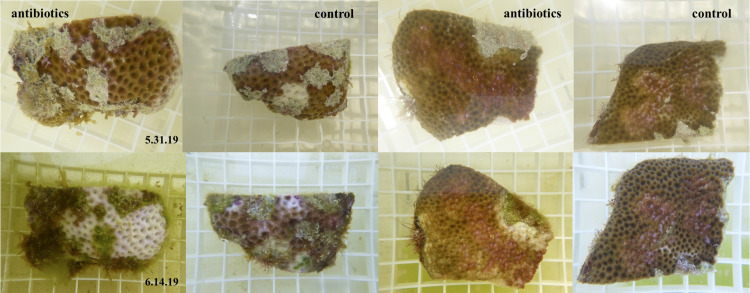
Representative photos of the response of diseased *S. siderea* fragments to antibiotic treatment. Coral fragments were split in half with one half treated with antibiotics and the other half left untreated as a control. Top row is of fragments at the start of the experiment and bottom row of fragments at the end of the experiment.

Initial lesion morphologies of the 18 diseased fragments used in this experiment included discolored patches (bleached or purple) with 61.1% having initial tissue loss and 27.8% without initial tissue loss, and 11.1% had tissue loss with no associated discoloration. During the experiment, 22% of the control fragments and 55.6% of the experimental fragments exposed to antibiotics had lesions that continued to progress (McNemar’s test, X^2^ = 0.67, p = 0.41). Otherwise, lesions appeared to re-pigment (heal) or showed no changes (stasis) for the duration of the study (Figs 10 and 11).

## Discussion

This study showed that lesions on the majority of tagged *S. siderea* colonies in the field progressed through time with associated chronic tissue loss. Most lesions had a slow rate of tissue loss resulting in partial but not total colony mortality. The case fatality rate during the 18-month study (Florida Keys: 5.9% and Fort Lauderdale: 0%) was low compared to other studies of coral diseases, especially stony coral tissue loss disease (SCTLD). As comparison, tagged *Montastraea cavernosa* colonies with SCTLD, at a nearby Florida Keys site, had a case fatality rate of 88.2% in the first year of the study and 21.4% in the 2^nd^ year of the study [32] and losses of up to 60% coral cover were found on SCTLD-affected reefs [7,36,37]. Other diseases can have a much lower degree of virulence. Gochfeld et al. [28] followed dark spot disease on *S. siderea* for two years and found no colony mortality and Aeby et al. [38] recorded a case fatality rate of 28% after two years for a chronic tissue loss disease in *Montipora capitata* in Hawaii.

Within the Florida Keys, initial lesion morphology on tagged *S. siderea* was important in determining disease outcome. Colonies with lesions, which at the start of the study, had discolorations (purple, brown or bleached) without any associated tissue loss had a much lower rate of colony mortality compared to lesions with some degree of tissue loss, no matter how small. Aeby et al. [32] also found that the initial lesion morphology in *Montastraea cavernosa* with SCTLD predicted disease outcome with lesions with bleached edges having significantly less tissue loss through time as compared to lesions without bleached margins. They hypothesized that the different lesion morphologies indicated changes in disease pathogenesis through time (different stages of the same disease). For *S. siderea*, most lesions changed gross morphologies and progressed to some degree of tissue loss by the end of the study leading us to hypothesize that, for colonies within the Florida Keys, most lesions were likely different stages of a single disease. It is interesting that within our study area at the Fort Lauderdale site we only found bleached lesions compared to the predominantly pigmented lesions (purple or brown) found within our site in the Florida Keys. *S. siderea* colonies with purple lesions do occur on Fort Lauderdale reefs, as two of the three samples collected for histology had purple lesions and were from the same Fort Lauderdale reef as our study site. However, when we were searching for colonies to sample for histology at Fort Lauderdale, we found that the number of *S. siderea* with purple lesions were scarce.

Lesion progression was slow in both regions but, in addition to differences in gross lesion morphologies of tagged colonies, the temporal pattern of prevalence differed. Lesions remained on colonies in the Florida Keys throughout the study whereas at Fort Lauderdale, the lesions in half of the colonies appeared to resolve through time. Sample sizes were small (n = 6) at Fort Lauderdale but there was a drop in disease in the summer months when only one colony showed disease signs. For other coral diseases, duration of disease on individual colonies ranges from chronic diseases such as growth anomalies [39] or *Porites* trematodiasis [40,41] to diseases which resolve and reappear on colonies such as dark spot disease [28], to diseases that are seasonal and associated with specific underlying environmental factors. For example, black band disease is more prevalent in warmer, summer months [42,43], and white syndrome outbreaks can occur in months with high rainfall and associated runoff [44,45] or with anomalously warm temperatures [46,47]. This highlights the complex nature of coral disease, and that each individual disease needs to be studied to understand disease pathogenesis. The bleached versus pigmented lesions found in each region could reflect different disease agents, or perhaps differences in environmental co-factors between regions.

Another possibility is that the differences in lesion morphology and pathogenesis reflect different stages of the same disease on reefs in the two regions. Disease pathogenesis can change through time, and the *S. siderea* disease could have emerged at different times on the reefs in these two regions, as occurred with the spread of SCTLD. Aeby et al. [32] compared disease dynamics of SCTLD-affected *Montastraea cavernosa* colonies in Fort Lauderdale where SCTLD emerged in 2014–2015 and the Florida Keys where SCTLD emerged in 2018. Tagged SCTLD-affected *M. cavernosa* colonies were followed between 2018 and 2020. At Fort Lauderdale, during both years of the study, colonies showed declining disease prevalence, low colony mortality, and disease lesions were mainly bleached spots without tissue loss. In contrast, in the first year of the study, Florida Keys colonies maintained 100% disease prevalence with high mortality, and disease lesions were predominantly tissue loss with no bleached edges. However, in year two, colonies in the Florida Keys showed declining disease prevalence, low mortality, and lesion morphology switched to a mixture of bleached polyps and tissue loss with or without bleached edges. They hypothesized that the change in SCTLD dynamics through time could be due to pathogen evolution, intrinsic differences among colonies in response to SCTLD, such that, the most susceptible colonies on a reef caught and died from the disease early on, or other co-factors not yet understood. It must be considered that a similar dynamic could be going on with the *S. siderea* disease in the two regions.

Alternatively, intraspecific variability in disease susceptibility or progression is not uncommon in corals and can be due to differences among colonies in the species of associated Symbiodiniaceae [48–50] or resident microbiota [51]. As such, the variability found in lesion morphologies, histology or disease pathogenesis could simply be due to intrinsic differences among *S. siderea* colonies. For *S. siderea*, another potential piece of the puzzle is the recent finding of three cryptic lineages of *S. siderea* [20,52]. The three lineages were found to differ in distribution, microbial associations, phenotypic traits of holobiont partners and skeleton morphologies. Future studies examining potential differences in disease susceptibilities among these cryptic lineages would prove valuable in understanding the underlying processes creating lesions in *S. siderea* in the Western Atlantic.

Histology samples were collected from coral colonies that had a wide spectrum of gross lesion morphologies. Sampled colonies had purple, brown or bleached lesions with different degrees of associated tissue loss ranging from minimal tissue loss on a small number of calices to colonies with almost complete tissue loss. Regardless of gross lesion morphology, all 16 histology sections of lesions from colonies had tissue necrosis (lytic or coagulative) with 13 also showing degradation or necrosis of the algal endosymbionts (Symbiodiniaceae), all of which indicates a disease process at the cellular level. Healthy reference samples obtained from the Dry Tortugas exhibited no tissue necrosis nor observable degradation of the endosymbionts. However, the cellular responses (histological signature) of affected coral colonies were slightly different between the three sample sites (Fort Lauderdale, Florida Keys: Looe Key, Haslun’s reef). There was variability in the proportion of colonies exhibiting noted histological parameters such as lytic necrosis, coagulative necrosis, degraded Symbiodiniaceae, Symbiodiniaceae with a positive periodic acid-Schiff (PAS) reaction (indicates starch storage in the symbiont) and coral acid rich proteins (CARP) indicating a host response to prevent endolithic invasions. The histological sample sizes were small, and unequal among sites but it does also suggest that differences in gross lesions on corals could be due to different environmental conditions or the temporal stage of the disease on individual colonies or reefs.

None of the histology samples showed infections of endolithic fungi which appears to rule out dark spot disease as a diagnosis for these lesions in *S. siderea*. Dark spot disease (DSD) also manifests as multi-focal or coalescing spots of dark discolored tissue and is found affecting 16 coral species including *S. siderea* [30]. Corals with similar disease signs found in the Indo-Pacific [53,54] and the Red Sea [10] are termed “endolithic hypermycosis” as histopathology showed the lesions to be associated with endolithic fungal infections. In the Caribbean, Renegar et al. [29] also found endolithic fungal infections in DSD-affected *S. siderea*. We did observe fungal hyphae in the skeletons of diseased specimens, but fungi are a known constituent of the microbial endolithic community in corals [55], and there were no cases where fungal hyphae penetrated coral tissues. DSD lesions are usually darker than the lesions on the colonies in our study, and disease progression also differed. Although DSD lesions can create a slow, progressive tissue loss [30], they more often resolve and appear elsewhere on the colony [18,28,56]. In addition, aquaria studies found no evidence of transmissibility in DSS-affected *S. siderea* [57].

Interestingly, endolithic algae were found in all samples examined from affected colonies but none of the healthy colonies from Dry Tortugas. Coral skeletons harbor a wide array of eukaryotic (microalgae and fungi) and prokaryotic (cyanobacteria, heterotrophic bacteria, and archaea) microbes which are thought to play a role in coral health and disease [58,59]. Endolithic algae can become more abundant with increased light availability such as when bleached corals lose their endosymbiotic Symbiodiniaceae [60,61]. Perhaps the lesions associated with this disease (bleached and pigmented tissues) allow more light to pass through the coral tissue and so are changing the dynamics between coral host and associated endoliths, and further study is warranted on this hypothesis. Endoliths are an important component of coral health, such as endolithic algae providing photoassimilates to coral during bleaching [62]. Despite their importance, coral endolithic communities are not well-studied and should be the focus of further research [58,63].

Aquaria studies showed that lesions in *S. siderea* were transmissible through direct contact and through the water column for both test species, *S. sidere*a and *O. faveolata*. Not surprising, transmission success was higher with direct contact in both test species, but lesions did also develop in non-touching fragments. These observations are consistent with the etiology of the chronic tissue loss disease in *S. siderea* being a communicable agent, but it must be kept in mind that these experiments were conducted under artificial conditions, which likely affected the rates of transmission. Therefore, although the conclusions drawn about whether the disease is transmissible or not are supported, the actual rates may differ from transmission occurring under natural conditions.

In the field, differences in gross lesion morphology translated into variable rates of disease progression and so we also examined the relationship between gross lesion morphology (with or without initial tissue loss) and transmission success in the aquaria trials. For each test species (*S. siderea* and *O. faveolata*), both types of lesions (discoloration with or without tissue loss) resulted in successful transmission (development of a lesion). However, lesions with initial tissue loss transmitted more successfully upon contact. None of the seven disease fragments with lesions (purple pigmentation or bleaching) without associated tissue loss transmitted successfully to *S. siderea* and only two out of the seven transmitted to *O. faveolata*. This contrasts with a transmission success of 36.4% and 63.6% respectively, to *S. siderea* and *O. faveolata* from lesions with some degree of tissue loss. Lesions with associated tissue loss may represented a disease in a more advanced state, different etiological agents or simply that, with an open wound, the disease agent was able to transmit more readily to the healthy fragment.

Aquaria studies also found that *S. siderea* developed tissue loss lesions when exposed to *C. natans* with SCTLD. Transmission success was high for touching (100%) and non-touching (80%) fragments after an average of 8.4 days and 7 days, respectively. These findings are consistent with numerous field surveys reporting SCTLD in *S. siderea* [6,15,64]. In contrast, in the aquaria studies with chronic tissue loss lesions in *S. siderea*, transmission was less successful with only 22.2% of the healthy *S. siderea* fragments developing lesions with direct contact and only 5.6% of the non-touching fragments. Time to infection was longer at an average of 13.5 days for touching and 8 days for non-touching. There were also differences in the gross morphology of the lesions that developed on test fragments of *S. siderea* when exposed to *C. natans* with SCTLD versus *S. siderea* with chronic tissue loss disease. *S. siderea*, exposed to SCTLD developed small tissue loss lesions with no changes in pigmentation (purple or bleaching). However, when exposed to chronic tissue loss disease, lesions presented as small areas of tissue loss with a bleached margin along the lesion. In one case, both the touching and non-touching test fragments developed whole fragment bleaching as well as small tissue loss lesions. Again, this suggests that the disease agent(s) underlying SCTLD may be different from what is creating chronic tissue loss lesions in *S. siderea*. These were both short-term aquaria trials and so might not represent normal disease pathogenesis in the field.

Regardless of gross lesion morphology, none of the lesions responded to treatment with the antibiotics, amoxicillin and kanamycin. This is in sharp contrast with studies on other coral species where SCTLD lesion progression was halted or slowed [11–13,65]. This offers more evidence suggesting that pigmented tissue loss lesions in *S. siderea* are different than SCTLD. It also suggests that bacterial pathogens may not be involved in the disease process, or at least, bacterial pathogens that are sensitive to amoxicillin or kanamycin. Future studies could employ different classes of antibiotics targeting other potential strains of bacterial pathogens. Interestingly, many of the lesions in the experiment did not progress and some even started to heal. Of course, these were short-term (maximum of 3 weeks) aquaria experiments but tagged colonies in the field with similar lesions did continue to progress through time. In experimental aquaria, fragments were held in clean, filter-sterilized water in contrast to the polluted waters found on Florida’s coral reefs [66]. It may be that there are environmental co-factors contributing to chronic tissue loss disease in *S. siderea* as has been found for numerous other coral diseases [44,45,67–70], including dark spot disease in *S. siderea* across Florida’s Coral Reef [31].

The current case definition of SCTLD stipulates microscopic evidence of lytic necrosis of gastrodermis of basal body wall and endosymbiont degeneration and necrosis [14]. Based on this definition, 13 of the 16 samples would be defined, microscopically, as SCTLD. However, Work et al. [71] examined corals with gross lesions consistent with SCTLD from the US Virgin Islands, which showed a different microscopy pathology. They found lytic mucus cell hypertrophy and necrosis with no involvement of mesoglea, and minimal endosymbiont pathology. Multiple diagnostic criteria should be employed when identifying any specific disease. Presence of gross lesions, disease virulence, host species affected, temporal patterns, as well as laboratory analyses including histology should all be taken into consideration. For *S. siderea*, lesion morphology, rate of tissue loss and case fatality was very different from other coral species affected by SCTLD and none of the lesions halted in response to antibiotic treatment. *Siderastrea siderea* does display tissue loss lesions consistent with SCTLD [15–17] and *S. sidera* did develop gross tissue loss lesions similar with other species during past outbreaks of acute tissue loss diseases such as white plaque type II in the Florida Keys in 1995 [72] and an outbreak of acute tissue loss disease in the Dry Tortugas, Fl [73]. Since, the histological signature of lesions was consistent with SCTLD we cannot rule out that the same disease agent(s) that cause SCTLD are also creating the gross lesions (bleached or purple patches, with or without tissue loss) on *S. siderea.* However, the other diagnostic criteria (lesion morphology, rate of tissue loss, overall colony mortality and response to antibiotics) were not consistent with SCTLD, and so SCTLD cannot be confirmed for these lesions on *S. siderea*. For field signs of coral disease, accepted disease nomenclature for potentially new lesions is based on a morphologic description of the lesion without inferring causation [9]. As such, until we know more about disease in *S. siderea*, we suggest that pigmented lesions with minimal tissue loss should be termed, *Siderastrea siderea* chronic tissue loss disease. Finally, it is noted that *S. sidera* with similar lesions (pigmentation with or without tissue loss) have been reported from many regions of the Caribbean [74–76], however, the pathogenesis and etiology of these lesions may differ between Florida and the Caribbean. Identifying coral diseases exclusively via field signs is unreliable and highlights the importance of incorporating multiple levels of analyses to understand coral diseases and their causes.

## Supporting information

S 1 Fig Pictures of gross lesions of *Siderastrea sidera* sampled for histology. Sixteen *S. siderea* colonies with lesions sampled for histology from three different reefs (Looe Key, Haslun’s Reef, Fort Lauderdale) along Florida’s Coral Reef.
(PDF)

S 1 Table Excel spreadsheet of gross and histopathological parameters. Histology was conducted on sixteen lesion samples taken from *S. siderea* colonies on three different reefs (Looe Key, Haslun’s Reef, Fort Lauderdale) along Florida’s Coral Reef.
(XLSX)
