# Supplementary figures and images for: Progressive chronic tissue loss disease in Siderastrea siderea on Florida’s coral reef
Source: PLoS One. 2025 Aug 6;20(8):e0329911. doi: 10.1371/journal.pone.0329911 (PMC12327659; doi:10.1371/journal.pone.0329911)

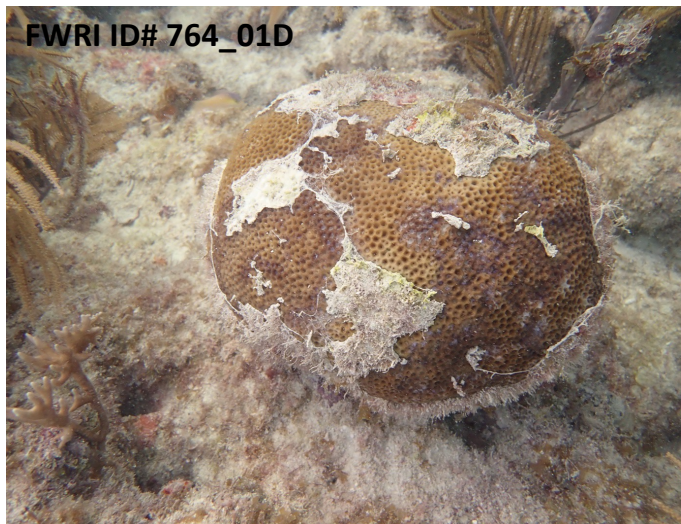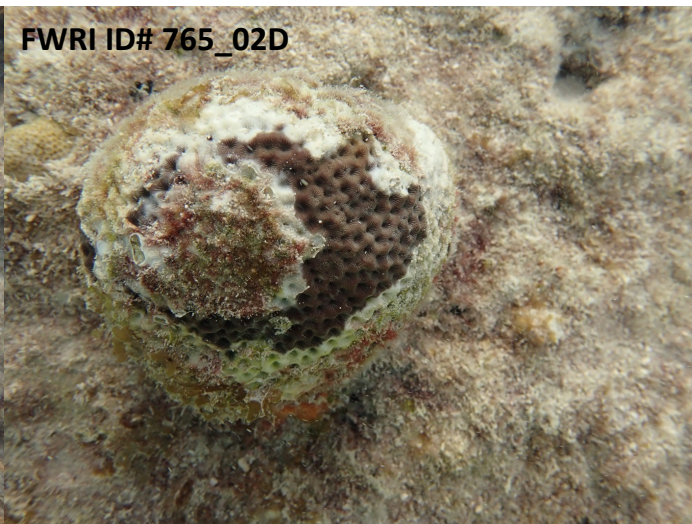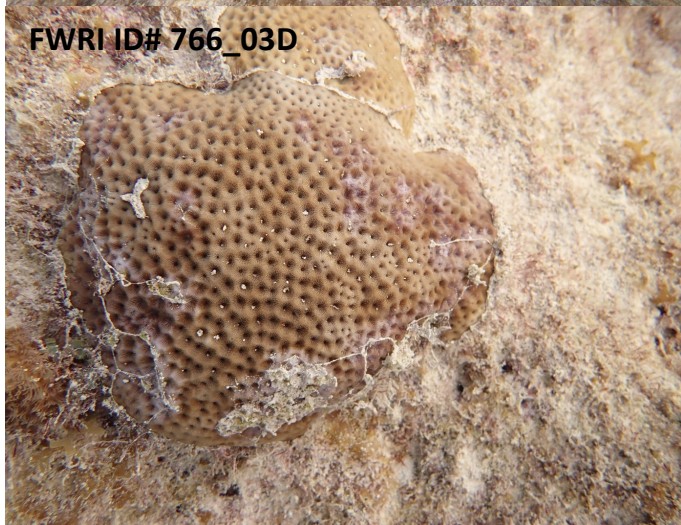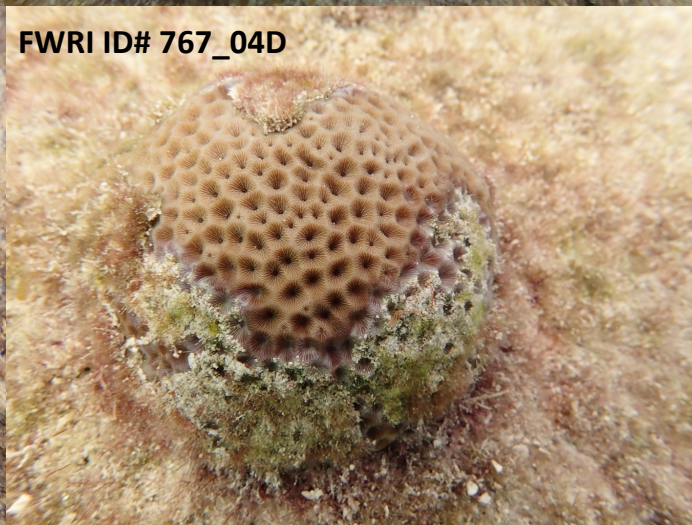

Looe Key

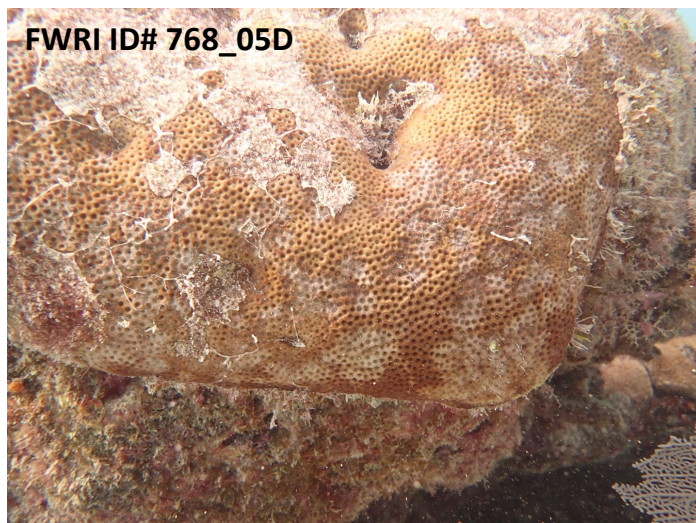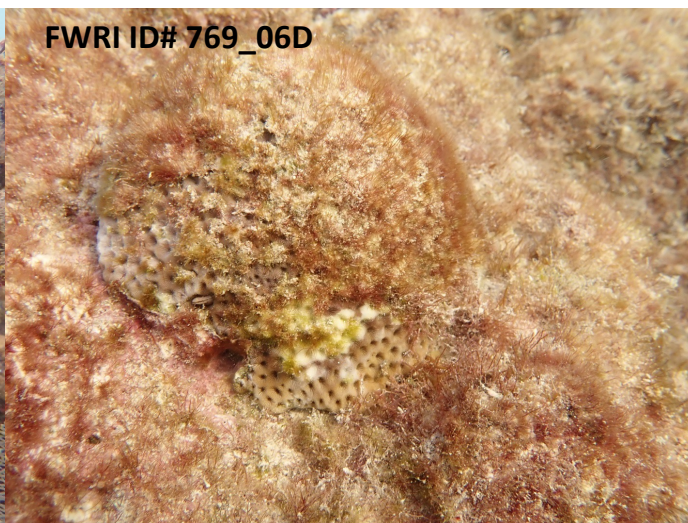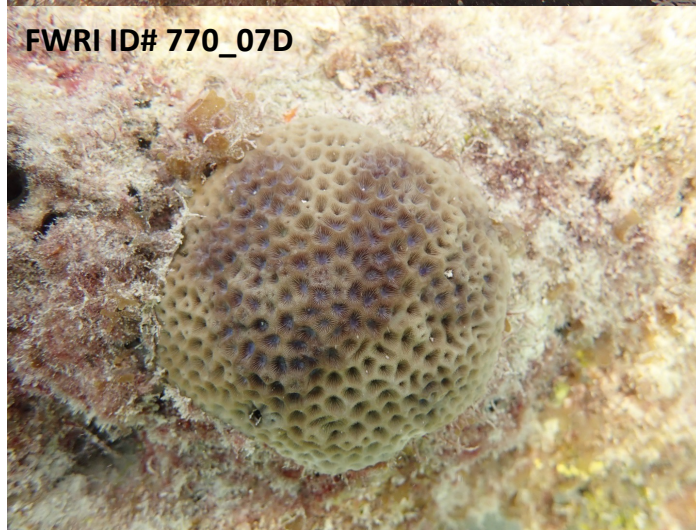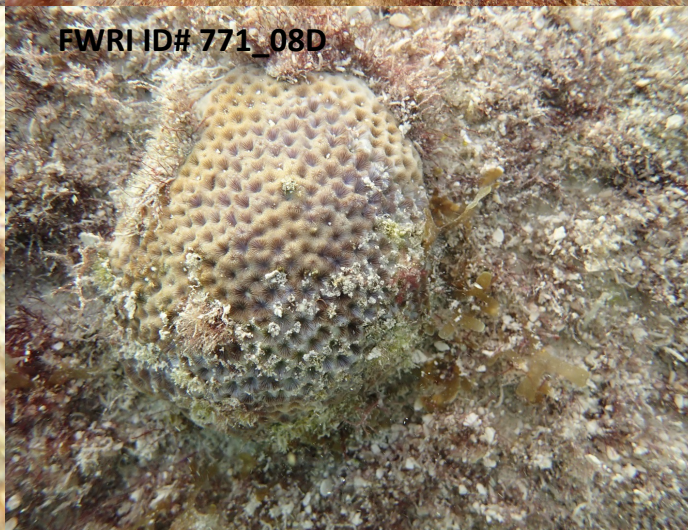

Looe Key

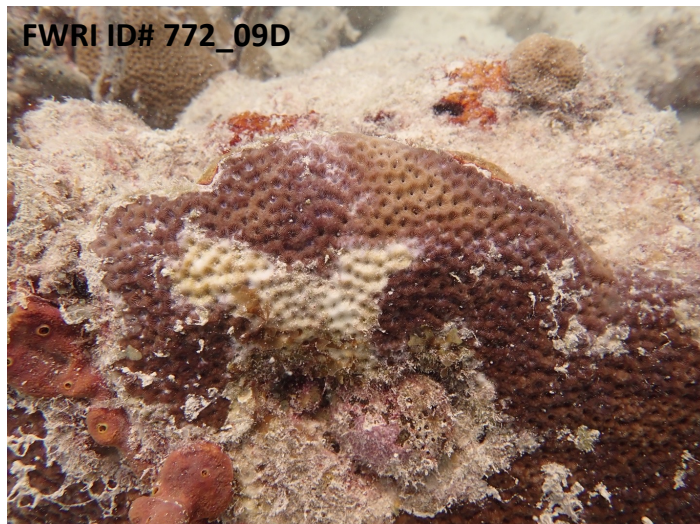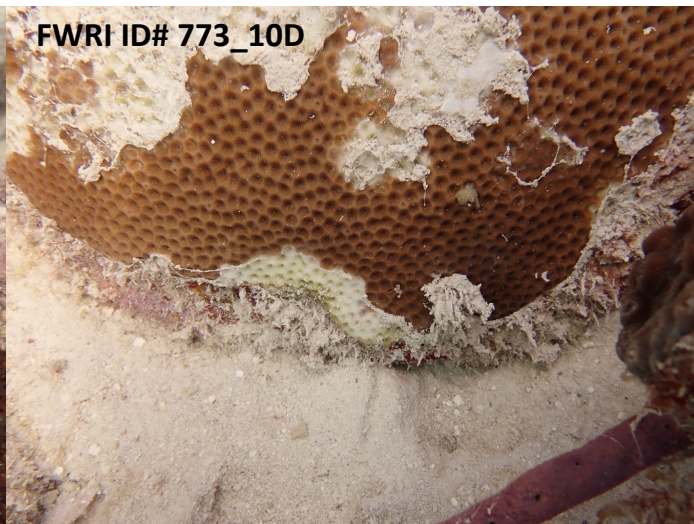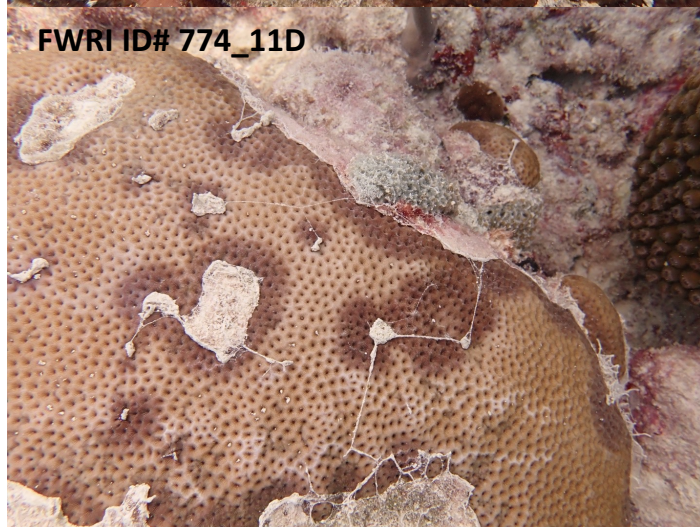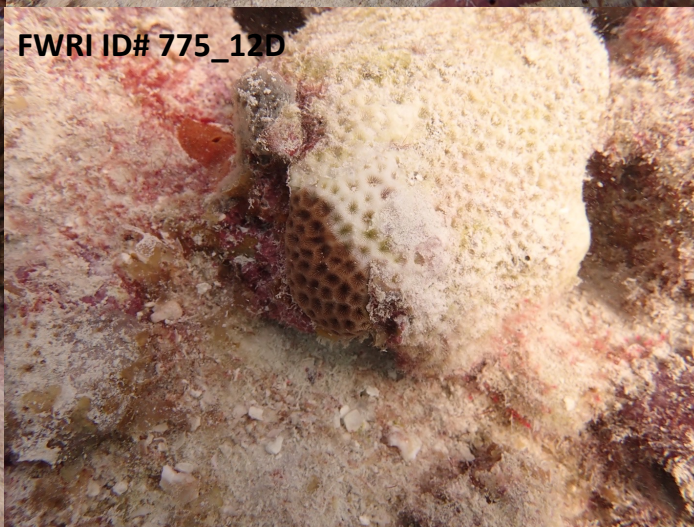

Haslun's Reef

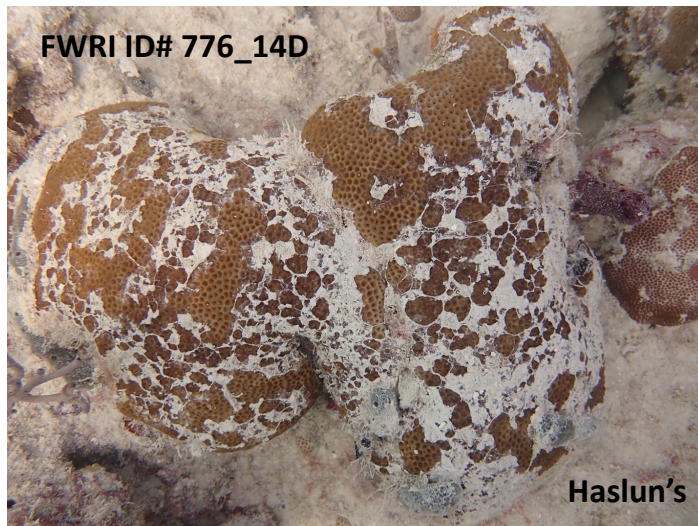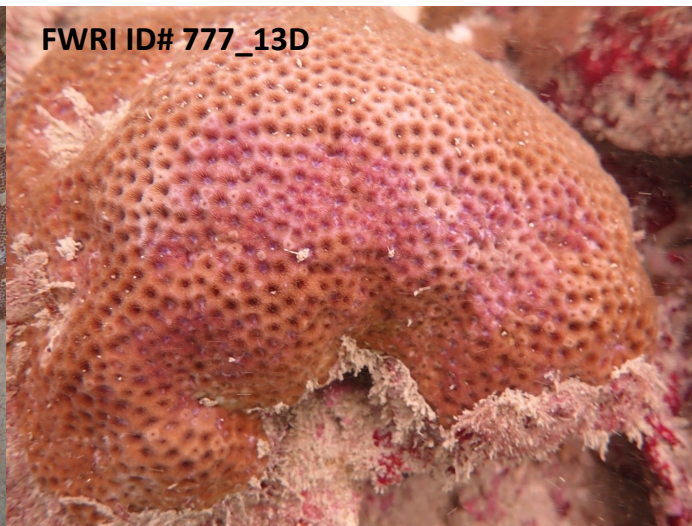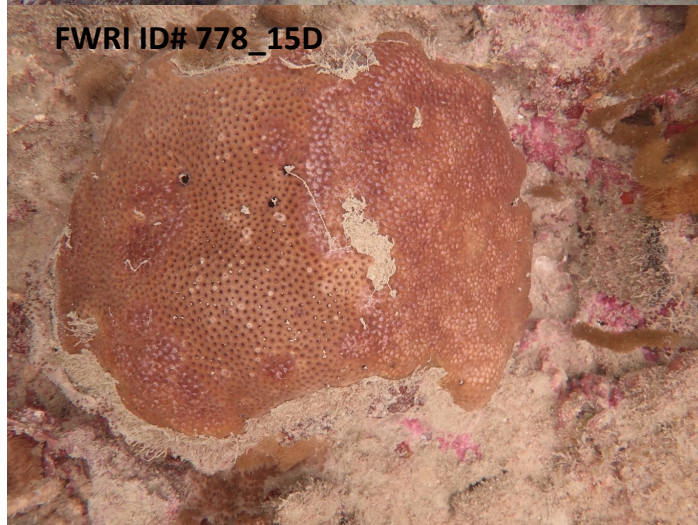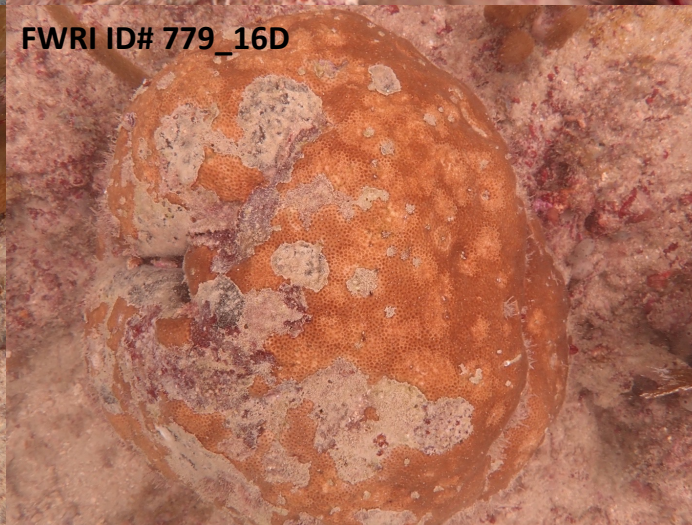

Fort Lauderdale

Supplement: S 1 Fig — Sixteen S. siderea colonies with lesions sampled for histology from three different reefs (Looe Key, Haslun’s Reef, Fort Lauderdale) along Florida’s Coral Reef. (PDF) [file pone.0329911.s001.pdf]
